# Intrathecal Pemetrexed Administration and Myelosuppression in Patients with Leptomeningeal Metastases from Lung Adenocarcinoma: A Retrospective Study

**DOI:** 10.32604/or.2025.064237

**Published:** 2025-07-18

**Authors:** Junxing Chen, Luping Pan, Yunzhi Liu, Yan Fang, Ruoxuan Li, Zhiqin Lu, Anwen Liu, Yanqing He, Zhimin Zeng

**Affiliations:** 1 Department of Oncology, The Second Affiliated Hospital of Nanchang University, Nanchang, 330006, China; 2 School of Public Health, Nanchang University, Nanchang, 330006, China; 3 Department of Radiotherapy, The First Affiliated Hospital of Zhejiang Chinese Medical University, Zhejiang Provincial Hospital of Traditional Chinese Medicine, Hangzhou, 310000, China; 4 Radiation Induced Heart Damage Institute of Nanchang University, The Second Affiliated Hospital, Jiangxi Medical College, Nanchang University, Nanchang, 330006, China; 5 Department of Nosocomial Infection Control, The Second Affiliated Hospital of Nanchang University, Nanchang, 330006, China; 6 Jiangxi Province Key Laboratory of Immunology and Inflammation, The Second Affiliated Hospital, Jiangxi Medical College, Nanchang University, Nanchang, 330006, China

**Keywords:** Leptomeningeal metastasis, lung adenocarcinoma, myelosuppression, intrathecal pemetrexed

## Abstract

**Background:**

Non-small cell lung cancer (NSCLC) patients with leptomeningeal metastasis (LM) have a very poor prognosis. Intrathecal pemetrexed (IP) has shown moderate efficacy in treating patients with NSCLC-LM. Myelosuppression is the most common adverse effect following IP administration. Despite this trend, the specific risk factors contributing to IP-related myelosuppression remain unclear.

**Methods:**

This study conducted a retrospective analysis of lung adenocarcinoma (LUAD) patients with LM who received IP treatment at the Second Affiliated Hospital of Nanchang University from April 2017 to April 2024. Risk factors for myelosuppression were identified through univariate and multivariate logistic regression analyses. Non-linear relationships and determined the inflection points were subsequently determined using smooth curve fitting and threshold effect analysis

**Results:**

A total of 95 patients were identified, among whom 64 (68.42%) experienced myelosuppression, with 43 (45.26%) cases classified as severe myelosuppression. Leukopenia emerged as the most prevalent form of myelosuppression. Age was established as an independent risk factor for both myelosuppression and its severe form. A nonlinear relationship between age and severe myelosuppression was observed. The risk of developing severe myelosuppression increased significantly with age, beyond the turning point of 58 years old (OR 1.28, 95% CI 1.08–1.52; *p* = 0.0042)

**Conclusions:**

Advanced age is associated with the occurrence of myelosuppression and severe myelosuppression. The probability of developing severe myelosuppression increases significantly in individuals aged 58 years or older

## 1 Introduction

Leptomeningeal metastasis (LM) is a severe complication of non-small cell lung cancer (NSCLC), characterized by the dissemination of tumor cells to the leptomeninges, including the pia mater, arachnoid membrane, subarachnoid space, and other cerebrospinal fluid (CSF) compartments [1,2]. LM occurs in approximately 3%–5% of patients with NSCLC, and its incidence has been increasing in recent years because of the extended survival period of cancer patients [3,4]. Otherwise, LM typically demonstrates poor response to conventional chemotherapy (CT) and radiotherapy, with a median overall survival (OS) of only 1 to 3 months [3]. However, for lung cancer patients with driver gene-positive LM who receive targeted therapy, the median OS extends to 3 to 11 months [3,5–7].

Previous studies have indicated a higher incidence of LM in lung adenocarcinoma (LUAD) patients with driver gene mutations, including 9.4% of patients with EGFR mutations and 10.3% of patients with ALK rearrangement [8,9]. Some studies have suggested that intrathecal pemetrexed (IP) exerts certain curative effects on these patients [10]. Prospective trials involving NSCLC patients with LM (NSCLC-LM) have demonstrated the safety and efficacy of IP, with response rates of 30%–70% and disease control rates of 50%–80% [11–13]; Notably, myelosuppression emerged as the most frequent adverse event in these studies [11–13]. Recently, Fan et al. reported an 84.6% response rate and a median OS of 9 months in 30 NSCLC-LM patients with EGFR mutations who did not respond to tyrosine kinase inhibitor (TKI) treatment with IP; moreover, myelosuppression was identified as the predominant side effect in these patients [14]. Subsequently, the authors expanded their phase II study to include 132 NSCLC-LM patients. The results of this trial indicated an 80% response rate and a median OS of 12 months, with 31.8% of patients experiencing myelosuppression; this finding further emphasized the prevalence of myelosuppression as a side effect [15]. Our group reported an IP response rate of 68.3% and a median OS of 10.1 months; additionally, consistent with other studies, myelosuppression was also identified as the most common adverse effect [16]. Collectively, these findings suggest that myelosuppression is the primary adverse event associated with IP treatment.

Myelosuppression resulting from intravenous CT is typically associated with several factors such as advanced age, poor performance status, comorbidities, female sex, impaired hepato-renal functions, low baseline white blood cell counts (WBC), low body mass index (BMI) or body surface area, and advanced disease stage [17–20]. However, the risk factors for myelosuppression, particularly severe cases, related to IP remain unclear. The present study aimed to investigate the association between IP and myelosuppression in patients with LM from LUAD (LUAD-LM).

## 2 Methods

### 2.1 Patients

This retrospective cohort study collected data from LUAD-LM patients treated at the Second Affiliated Hospital of Nanchang University between April 2017 and April 2024. The study adhered to the STROBE guidelines, by following the 22-item checklist for transparent and rigorous reporting, and was approved by the Ethics Committee of the Second Affiliated Hospital of Nanchang University. The ethics committee waived off the requirement for informed consent because of the retrospective nature of the study. LM was diagnosed based on clinical suspicion according to the European Association of Neuro-Oncology and European Society for Medical Oncology criteria and confirmed through positive imaging and/or CSF findings [21]. IP was defined as the administration of pemetrexed through lumbar puncture or an Ommaya reservoir. Myelosuppression was evaluated according to the Common Terminology Criteria for Adverse Events version 5.0. The specific criteria are as follows: (1) leukopenia is graded by WBC count as follows: Grade I (≥3.0 but <4.0 × 10^9^/L), Grade II (≥2.0 but <3.0 × 10^9^/L), Grade III (≥1.0 but <2.0 × 10^9^/L), and Grade IV (<1.0 × 10^9^/L); (2) neutropenia is graded by absolute neutrophil count: Grade I (≥1.5 but <2.0 × 10^9^/L), Grade II (≥1.0 but <1.5 × 10^9^/L), Grade III (≥0.5 but <1.0 × 10^9^/L), and Grade IV (<0.5 × 10^9^/L); and (3) thrombocytopenia is graded by platelet count: Grade I (≥75 but <100 × 10^9^/L), Grade II (≥50 but <75 × 10^9^/L), Grade III (≥25 but <50 × 10^9^/L), and Grade IV (<25 × 10^9^/L). Inclusion criteria were as follows: (1) pathologically confirmed LUAD; (2) LM confirmed by radiographical and/or CSF pathological examination; (3) at least one intrathecal CT session; and (4) baseline WBC count >3.5 × 10^9^/L, neutrophil count >2 × 10^9^/L, and platelet count >100 × 10^9^/L. Exclusion criteria were as follows: (1) patients with concurrent malignancies other than LUAD and (2) use of intrathecal CT drugs other than pemetrexed.

### 2.2 Data Collection

Clinical data, including demographic information, clinical characteristics, tumor-related features, treatment modalities, and clinical outcomes, were extracted from the electronic medical record database. The study analyzed variables potentially associated with myelosuppression, including sex, age, smoking history, Eastern Cooperative Oncology Group Performance Status (ECOG PS) score at LM diagnosis, timing of LM diagnosis, presence of bone metastasis (BM) and brain metastasis (BMs) at LM diagnosis, and hematological parameters (WBC count, neutrophil count, and platelet count) at myelosuppression occurrence. Treatment information and clinical outcomes included the timing and cycle of intrathecal injections, time to myelosuppression occurrence, systemic treatment pre- and post-LM diagnosis, and date of death or last follow-up.

### 2.3 IP Administration

IP was primarily administered through lumbar puncture or an Ommaya reservoir. Prior to IP administration, patients received an intramuscular injection of 1000 μg of vitamin B12, followed by vitamin B12 injections every 3 weeks, and daily oral administration of 400 μg of folic acid. The IP procedure involved pretreatment with 5 mg of dexamethasone, followed by the administration of pemetrexed. Based on our previous research and other studies [12,13,15,16], the dosing schedule and treatment cycle were as follows: (1) induction therapy: 10 mg of pemetrexed administered twice weekly for 2 weeks, (2) consolidation therapy: 10–30 mg, with some doses at 50 mg, administered weekly for 4 weeks, and (3) maintenance therapy: 10–30 mg administered every 3–4 weeks.

### 2.4 Statistical Analysis

Continuous variables were analyzed using the *t*-test for normally distributed data or the Kruskal-Wallis test for non-normally distributed data, while categorical variables were assessed using the chi-square test. Descriptive statistics were used to characterize myelosuppression occurrence, with rates reported for various levels and types of myelosuppression. Additionally, medians with interquartile ranges were provided to indicate the number of IP cycles at the onset of myelosuppression.

Univariate analyses were conducted to identify potential variables associated with myelosuppression. Variables with a *p*-value of <0.5 in univariate analysis were incorporated into multivariate regression analysis, which identified age as a risk factor for both myelosuppression and severe myelosuppression. Subsequently, the relationship between age and severe myelosuppression was examined using a smoothing plot, following adjustment for potential confounders. A two-piecewise linear regression model was employed to investigate the threshold effect of age on severe myelosuppression based on the smoothing plot. The threshold age at which the relationship between age and severe myelosuppression attained significance was determined using an iterative method, where the inflection point was adjusted within a predefined interval to maximize model likelihood. A two-tailed *p* value of <0.05 was considered statistically significant. All statistical analyses were conducted using R program (http://www.R-project.org).

## 3 Results

### 3.1 Patient Characteristics

**Figure 1:**
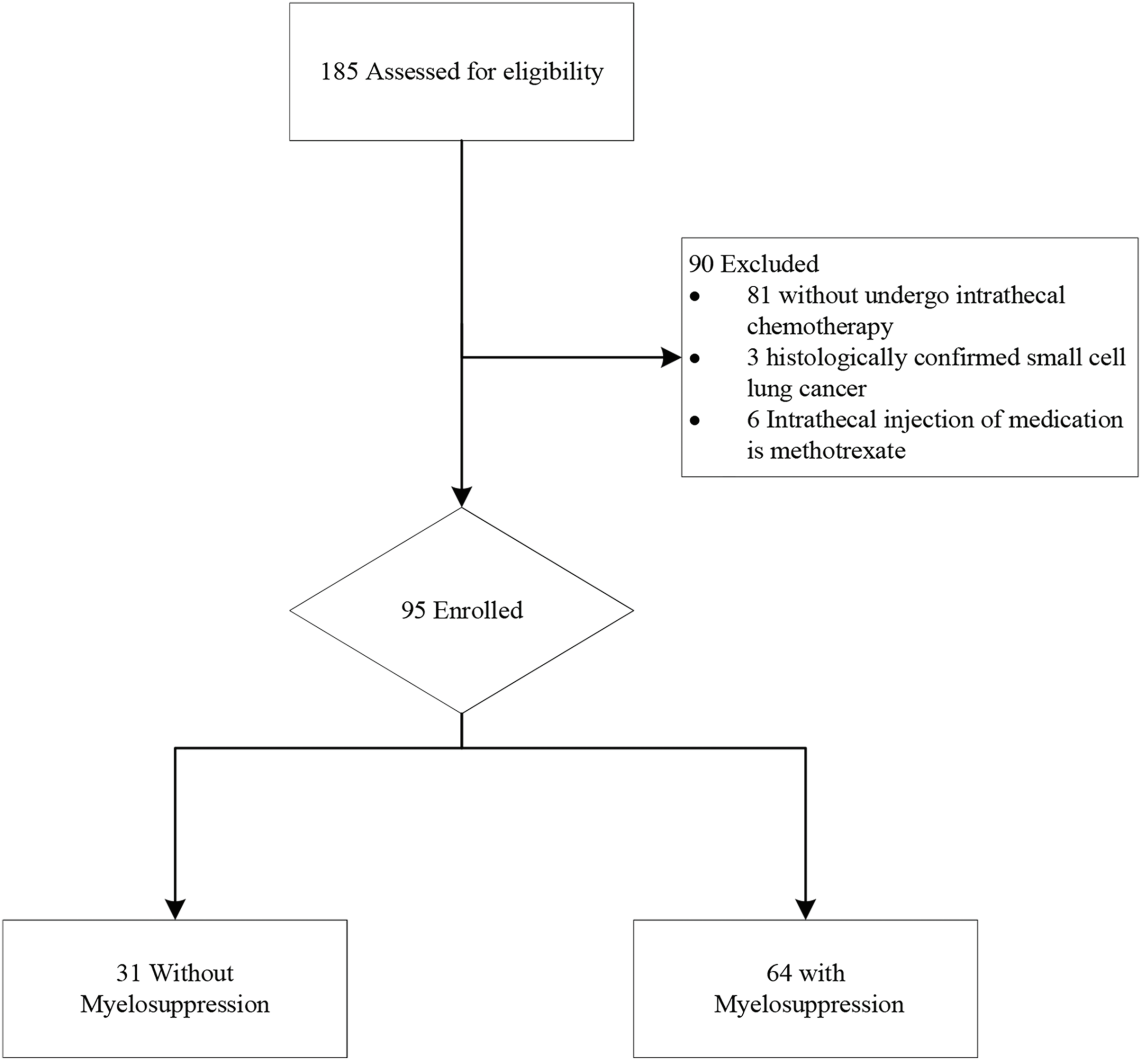
Study flow chart of patient selection

The study included the data of 185 patients diagnosed to have lung cancer and LM from the electronic medical record system between April 2017 and April 2024. A flowchart of the patient screening process is illustrated in Fig. 1.

Of the 95 patients enrolled in this study, the median follow-up time was 7.43 months. Baseline clinical and demographic characteristics are summarized in Table 1. Fifty-two patients were women (54.74%) and 43 patients were men (45.26%). The mean (SD) age of the patients was 57.43 (8.58) years. The mean (SD) BMI of the patients at LM diagnosis was 21.69 (3.39) kg/m². The majority of patients were positive for driver gene mutations at the initial diagnosis. Prior to the diagnosis of LM, 35 (36.84%) patients received intravenous CT, with only 16 (16.84%) patients receiving beyond second-line treatment. At LM diagnosis, 71 patients (74.74%) had BMs, 48 patients (50.53%) had BM, and 8 patients (8.42%) had an ECOG PS > 2. Seventy-six patients (80.00%) received TKI therapy, 22 patients (23.16%) underwent intravenous CT, 11 patients (11.58%) received whole brain radiotherapy (WBRT), and 58 patients (61.05%) received antiangiogenic therapy. Regarding the IP administration route, 74 (77.89%) patients received medication through lumbar puncture.

**Table 1:** Characteristics of the study patients

		Myelosuppression			Severe myelosuppression		
	Total	Without	With	*p* value	Without	With	*p* value
**Number**	95	31	64		52	43	
**Sex, n (%)**				0.670			0.065
Female	52 (54.74)	16 (51.61)	36 (56.25)		24 (46.15)	28 (65.12)	
Male	43 (45.26)	15 (48.39)	28 (43.75)		28 (53.85)	15 (34.88)	
**Age, Mean (SD)**	57.43 (8.58)	53.55 (7.54)	59.31 (8.48)	0.002*	55.44 ± 7.26	59.84 ± 9.49	0.012*
**Smoking, n (%)**				0.778			0.299
No	75 (78.95)	25 (80.65)	50 (78.12)		39 (75.00)	36 (83.72)	
Yes	20 (21.05)	6 (19.35)	14 (21.88)		13 (25.00)	7 (16.28)	
**ECOG PS, n (%)**				0.382			0.163
≤2	87 (91.58)	30 (96.77)	57 (89.06)		50 (96.15)	37 (86.05)	
>2	8 (8.42)	1 (3.23)	7 (10.94)		2 (3.85)	6 (13.95)	
**BMI, Mean (SD)**	21.69 ± 3.39	22.08 ± 4.04	21.50 ± 3.05	0.307	22.01 ± 3.71	21.34 ± 2.98	0.359
**Mutaion type initial, n (%)**			0.548			0.652	
Wild type	13 (13.68)	5 (16.13)	8 (12.50)		8 (15.38)	5 (11.63)	
EGFR 19del	33 (34.74)	10 (32.26)	23 (35.94)		20 (38.46)	13 (30.23)	
EGFR L858R	36 (37.89)	10 (32.26)	26 (40.62)		17 (32.69)	19 (44.19)	
ALK	1 (1.05)	1 (3.23)	0 (0.00)		1 (1.92)	0 (0.00)	
Others	12 (12.63)	5 (16.13)	7 (10.94)		6 (11.54)	6 (13.95)	
**CT before LM, n (%)**			0.793			0.431	
No	60 (63.16)	19 (61.29)	41 (64.06)		31 (59.62)	29 (67.44)	
Yes	35 (36.84)	12 (38.71)	23 (35.94)		21 (40.38)	14 (32.56)	
**Treatment lines before LM, n (%)**			0.104			0.494	
≤2	79 (83.16)	23 (74.19)	56 (87.50)		42 (80.77)	37 (86.05)	
>2	16 (16.84)	8 (25.81)	8 (12.50)		10 (19.23)	6 (13.95)	
**BMs at LM, n (%)**				0.154			0.311
No	24 (25.26)	5 (16.13)	19 (29.69)		11 (21.15)	13 (30.23)	
Yes	71 (74.74)	26 (83.87)	45 (70.31)		41 (78.85)	30 (69.77)	
**BM at LM, n (%)**				0.558			0.910
No	47 (49.47)	14 (45.16)	33 (51.56)		26 (50.00)	21 (48.84)	
Yes	48 (50.53)	17 (54.84)	31 (48.44)		26 (50.00)	22 (51.16)	
**IP administration, n (%)**			0.545			0.802	
Intrathecal injection	74 (77.89)	23 (74.19)	51 (79.69)		40 (76.92)	34 (79.07)	
Ommaya reservoir	21 (22.11)	8 (25.81)	13 (20.31)		12 (23.08)	9 (20.93)	
**WBRT after LM, n (%)**			0.951			0.758	
No	84 (88.42)	28 (90.32)	56 (87.50)		45 (86.54)	39 (90.70)	
Yes	11 (11.58)	3 (9.68)	8 (12.50)		7 (13.46)	4 (9.30)	
**CT after LM, n (%)**			0.541			0.137	
No	73 (76.84)	25 (80.65)	48 (75.00)		43 (82.69)	30 (69.77)	
Yes	22 (23.16)	6 (19.35)	16 (25.00)		9 (17.31)	13 (30.23)	
**Antiangiogenic after LM, n (%)**			0.023*			0.026*	
No	37 (38.95)	7 (22.58)	30 (46.88)		15 (28.85)	22 (51.16)	
Yes	58 (61.05)	24 (77.42)	34 (53.12)		37 (71.15)	21 (48.84)	
**ICI after LM, n (%)**			0.626			0.855	
No	89 (93.68)	28 (90.32)	61 (95.31)		48 (92.31)	41 (95.35)	
Yes	6 (6.32)	3 (9.68)	3 (4.69)		4 (7.69)	2 (4.65)	
**TKI after LM, n (%)**			0.126			0.18	
No	19 (20.00)	9 (29.03)	10 (15.62)		13 (25.00)	6 (13.95)	
Yes	76 (80.00)	22 (70.97)	54 (84.38)		39 (75.00)	37 (86.05)	

Note: n: number, SD: standard deviation, ECOG PS: Eastern Cooperative Oncology Group Performance Status score, BMI: body mass index, BMs: brain metastases, BM: bone metastases, LM: leptomeningeal metastasis, EGFR: epidermal growth factor receptor, ALK: anaplastic lymphoma kinase, WBRT: whole-brain radiotherapy, CT: chemotherapy, ICI: immune checkpoint inhibitors, TKI: tyrosine kinase inhibitor. *p < 0.05 indicates statistical significance.

### 3.2 Profile of Myelosuppression after Intrathecal Pemetrexed

A major proportion of the patients (64/95, 67.37%) experienced myelosuppression during IP treatment, with 43 patients (45.26%) developing severe myelosuppression (Table 2). The distribution of myelosuppression grades 0 to 4 is detailed in Table 2. Leukopenia, observed in 82.81% of the patients, was the most prevalent cytopenia associated with myelosuppression, followed by neutropenia (78.13%) and thrombocytopenia (67.19%). These findings are consistent with the hematological profile observed in severe myelosuppression. The median number of IP cycles required to induce myelosuppression was 4 (range: 2–5), while the median number of IP cycles for severe myelosuppression development was 3 (range: 2–4.5).

**Table 2:** Distribution of myelosuppression

Myelosuppression		Severe myelosuppression	
Grade 0	31 (32.63%)	≤Grade 2	52 (54.74%)
Grade 1	3 (3.16%)		
Grade 2	18 (18.95%)		
Grade 3	16 (16.84%)	≥Grade 3	43 (45.26%)
Grade 4	27 (28.42%)		
**Types of myelosuppression**		**Types of myelosuppression**	
Leukopenia	53 (82.81%)	Leukopenia	31 (72.09%)
Neutropenia	50 (78.13%)	Neutropenia	29 (67.44%)
Thrombocytopenia	43 (67.19%)	Thrombocytopenia	24 (55.81%)
**Cycles of IP**		**Cycles of IP**	
With myelosuppression	4.00 (2.00–5.00)	With severe myelosuppression	3.00 (2.00, 4.50)
Without myelosuppression	5.50 (3.00–11.50)	Without severe myelosuppression	4.00 (3.00–7.25)

Note: IP: intrathecal pemetrexed.

### 3.3 Univariate and Multivariate Analyses of Myelosuppression and Severe Myelosuppression

To evaluate the factors associated with myelosuppression and severe myelosuppression in our cohort, we conducted univariate and multivariate analyses. In the univariate logistic regression analysis (Table 3), age was independently associated with an increased risk of myelosuppression (OR: 1.09; 95% CI: 1.03–1.16; *p* = 0.003) and severe myelosuppression (OR: 1.07; 95% CI: 1.02–1.13; *p* = 0.0098). In contrast, antiangiogenic therapy after LM was associated with a decreased risk of myelosuppression (OR: 0.27; 95% CI: 0.10–0.76; *p* = 0.0128) and severe myelosuppression (OR: 0.39; 95% CI: 0.17–0.90; *p* = 0.028).

**Table 3:** Univariate analysis for myelosuppression and severe myelosuppression

		Myelosuppression		Severe myelosuppression	
	Statistics	OR (95**%** CI)	*p* value	OR (95**%** CI)	*p* value
**Sex**					
Female	52 (54.74%)	Refrence		Refrence	
Male	43 (45.26%)	0.92 (0.39, 2.19)	0.8519	0.55 (0.24, 1.25)	0.1532
**Age, year**	57.43 ± 8.58	1.09 (1.03, 1.16)	0.0030*	1.07 (1.02, 1.13)	0.0098*
**Smoking**					
No	75 (78.95%)	Refrence		Refrence	
Yes	20 (21.05%)	1.10 (0.38, 3.21)	0.8643	0.76 (0.28, 2.08)	0.5952
**BM**					
No	47 (49.47%)	Refrence		Refrence	
Yes	48 (50.53%)	0.85 (0.36, 2.02)	0.7102	1.24 (0.55, 2.79)	0.5997
**ECOG PS**					
≤2	87 (91.58%)	Refrence		Refrence	
>2	8 (8.42%)	3.50 (0.41, 29.81)	0.2517	4.05 (0.77, 21.23)	0.0975
**BMI, kg/m** ^**2**^	21.69 ± 3.39	0.93 (0.81, 1.07)	0.3053	0.95 (0.83, 1.07)	0.3909
**CT before LM**					
No	60 (63.16%)	Reference		Reference	
Yes	35 (36.84%)	0.89 (0.37, 2.15)	0.793	0.71 (0.31, 1.66)	0.432
**Treatment lines before LM**					
≤2	79 (83.16%)	Reference		Reference	
>2	16 (16.84%)	0.41 (0.14, 1.23)	0.111	0.68 (0.23, 2.06)	0.495
**BMs**					
No	24 (25.26%)	Refrence		Refrence	
Yes	71 (74.74%)	0.48 (0.16, 1.45)	0.1958	0.49 (0.19, 1.26)	0.1403
**IP administration**					
Intrathecal injection	74 (77.89%)	Refrence		Refrence	
Ommaya reservoir	21 (22.11%)	0.73 (0.27, 2.01)	0.546	0.88 (0.33, 2.35)	0.802
**WBRT after LM**					
No	84 (88.42%)	Refrence		Refrence	
Yes	11 (11.58%)	1.26 (0.31, 5.14)	0.7443	0.66 (0.18, 2.42)	0.5304
**CT after LM**					
No	73 (76.84%)	Refrence		Refrence	
Yes	22 (23.16%)	1.31 (0.45, 3.76)	0.6207	2.07 (0.79, 5.46)	0.1412
**Antiangiogenic therapy after LM**					
No	37 (38.95%)	Refrence		Refrence	
Yes	58 (61.05%)	0.27 (0.10, 0.76)	0.0128*	0.39 (0.17, 0.90)	0.0280*
**ICI after LM**					
No	89 (93.68%)	Refrence		Refrence	
Yes	6 (6.32%)	0.92 (0.16, 5.31)	0.9239	0.59 (0.10, 3.36)	0.5482
**TKI after LM**					
No	19 (20.00%)	Refrence		Refrence	
Yes	76 (80.00%)	1.79 (0.63, 5.03)	0.2734	2.06 (0.71, 5.97)	0.1856

Note: OR: odds ratio, CI: confidence interval, BM: bone metastases, ECOG PS: Eastern Cooperative Oncology Group Performance Status score, BMI: body mass index, BMs: brain metastases, LM: leptomeningeal metastasis, WB RT: whole-brain radiotherapy, CT: chemotherapy, ICI: immune checkpoint inhibitors, TKI: tyrosine kinase inhibitor. *p < 0.05 indicates statistical significance.

Subsequently, variables with a *p* value of <0.5 in the univariate analysis were incorporated into a multivariate logistic regression model. Stepwise adjustments were applied for age, sex, ECOG PS, BMs, BMI, BM, CT after LM, antiangiogenic therapy after LM, and TKI after LM. The multivariate analysis identified age as a significant risk factor for myelosuppression (OR: 1.11; 95% CI: 1.03–1.19; *p* = 0.0053) (Table A1). Additionally, age (OR: 1.09; 95% CI: 1.02–1.16; *p* = 0.0085), CT after LM (OR: 5.90; 95% CI: 1.46–23.88; *p* = 0.0129), and ECOG PS > 2 (OR: 19.69; 95% CI: 1.76–220.03; *p* = 0.0155) were identified as risk factors for severe myelosuppression (Table 4).

**Table 4:** Multivariate analysis for severe myelosuppression

Exposure	Non-adjusted		Adjust I		Adjust II	
	OR (95% CI)	*p*-value	OR (95% CI)	*p*-value	OR (95% CI)	*p*-value
**Age, year**	1.09 (1.02, 1.16)	0.0096*	1.09 (1.02, 1.16)	0.0085*	1.09 (1.02, 1.16)	0.0085*
**BMI, kg/m** ^**2**^	0.86 (0.73, 1.03)	0.0942	0.89 (0.75, 1.06)	0.1946	0.89 (0.75, 1.06)	0.1946
**BM**						
No	Refrence		Refrence		Refrence	
Yes	1.08 (0.37, 3.17)	0.8880	1.02 (0.34, 3.05)	0.9744	1.02 (0.34, 3.05)	0.9744
**ECOG PS**						
≤2	Refrence		Refrence		Refrence	
>2	21.79 (1.86, 255.52)	0.0142*	19.69 (1.76, 220.03)	0.0155*	19.69 (1.76, 220.03)	0.0155*
**BMs**						
No	Refrence		Refrence		Refrence	
Yes	0.48 (0.14, 1.65)	0.2431	0.51 (0.15, 1.79)	0.2948	0.51 (0.15, 1.79)	0.2948
**CT after LM**						
No	Refrence		Refrence		Refrence	
Yes	5.23 (1.33, 20.56)	0.0179*	5.90 (1.46, 23.88)	0.0129*	5.90 (1.46, 23.88)	0.0129*
						
**Antiangiogenic therapy after LM**						
No	Refrence		Refrence		Refrence	
Yes	0.25 (0.08, 0.75)	0.0131*	0.23 (0.07, 0.72)	0.0114*	0.23 (0.07, 0.72)	0.0114*
**TKI after LM**						
No	Refrence		Refrence		Refrence	
Yes	5.39 (1.03, 28.13)	0.0455	5.15 (0.97, 27.38)	0.0545	5.15 (0.97, 27.38)	0.0545
**WBRT after LM**						
No	Refrence		Refrence		Refrence	
Yes	0.60 (0.13, 2.77)	0.5137	0.60 (0.13, 2.84)	0.5177	0.60 (0.13, 2.84)	0.5177

Note: OR: odds ratio, CI: confidence interval, BMI: body mass index, BM: bone metastases, ECOG PS Eastern Cooperative Oncology Group Performance Status score, BMs: brain metastases, LM: leptomeningeal metastasis, CT: chemotherapy, TKI: tyrosine kinase inhibitor, WBRT: whole-brain radiotherapy, Model Ⅰ adjusted for age and sex. Model Ⅱ adjusted for age, sex, BMI, ECOG PS, BMs, BM, CT after LM, antiangiogenic therapy after LM, and TKI after LM. *p < 0.05 indicates statistical significance.

### 3.4 Smooth Curve Fitting and Threshold Effect Analysis

**Figure 2:**
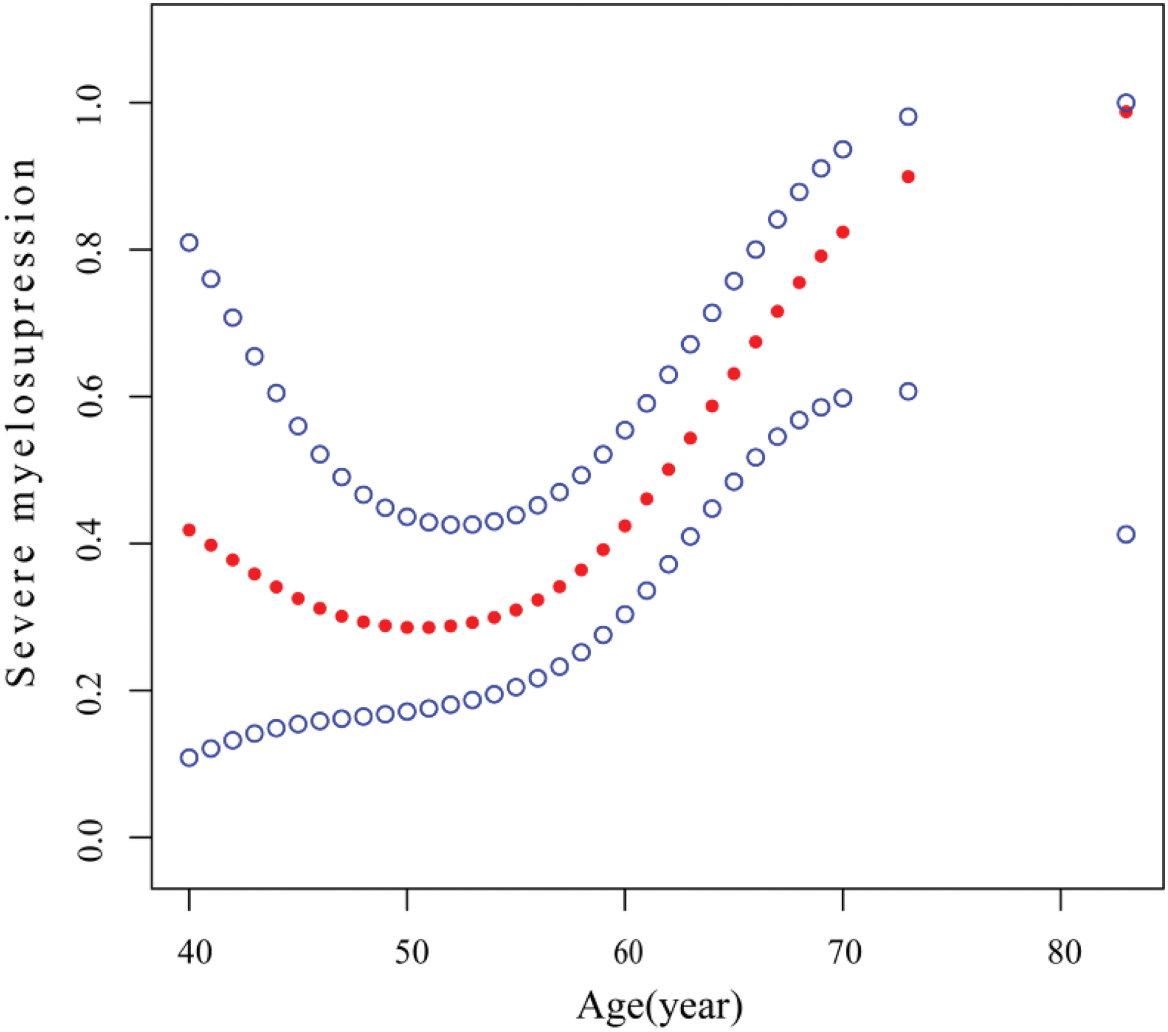
Association between age and severe myelosuppression. A threshold, nonlinear association between age and severe myelosuppression was identified (*p* = 0.023) using a generalized additive model. The solid red line represents the smooth curve fit between variables, and the blue bands indicate the 95% confidence interval of the fit. All results were adjusted for smoking status, sex, BM, ECOG PS, BMI, BMs, WBRT after LM, antiangiogenic therapy after LM, and TKI after LM. Abbreviations: BM: bone metastases, ECOG PS: Eastern Cooperative Oncology Group Performance Status score, BMI: body mass index, BMs: brain metastases, WBRT: whole-brain radiotherapy, LM: leptomeningeal metastasis, TKI: tyrosine kinase inhibitor

Our analysis revealed that age is associated with both myelosuppression and severe myelosuppression, which can significantly impact treatment outcomes and potentially pose a life-threatening risk [22–25]. To further investigate this relationship, we conducted curve fitting and threshold effect analysis to examine the association between age and severe myelosuppression. After adjusting for potential confounding factors—including smoking status, sex, BM, ECOG PS, BMI, BMs, WBRT, CT after LM, antiangiogenic therapy after LM, and TKI after LM— a smoothing curve fitting demonstrated a nonlinear relationship between age and severe myelosuppression following IP (Fig. 2). Beyond the turning point age of 58 years (OR: 1.28; 95% CI: 1.08–1.52; *p* = 0.0042), the risk of developing severe myelosuppression increased with age (Table 5). This finding suggests that severe myelosuppression is associated with age, with an elevated risk observed in patients aged 58 years or older.

**Table 5:** Threshold effect analysis of age on severe myelosuppression by using piecewise linear regression

Inflection points of age (year)	OR (95% CI)^a^	*p* value
<58	0.97 (0.86, 1.09)	0.5536
≥58	1.28 (1.08, 1.52)	0.0042*

Note: CI: confidence interval, OR: odds ratio, ^a^ Adjusted: BMI, ECOG PS, BM, CT after LM, antiangiogenic therapy after LM, TKI after LM, and WBRT after LM, *p < 0.05 indicates statistical significance.

## 4 Discussion

This study investigates the incidence and characteristics of myelosuppression in LUAD-LM patients treated with IP and the risk factors associated with the development of myelosuppression and severe myelosuppression. The findings revealed that a major proportion of patients (67.37%) experienced myelosuppression following IP treatment, with a considerable number of patients (45.26%) developing severe myelosuppression. Notably, the study found that age ≥58 years is a risk factor for severe myelosuppression. To the best of our knowledge, this study represents the first comprehensive examination of risk factors for myelosuppression in LUAD-LM patients undergoing IP treatment.

Previous prospective and retrospective studies have demonstrated that myelosuppression after IP is a common adverse effect [12–14,26,27]. However, the incidence of myelosuppression in previous studies typically ranged from 30% to 40% [12–15], whereas this study reported an incidence as high as 68.42%. Notably, apart from the study of Pan et al., which incorporated involved-field radiotherapy alongside IP treatment, the other three studies only evaluated IP treatment and/or its combination with TKI therapy. In contrast, our study included a major proportion of patients who, following LM diagnosis, received additional systemic therapies—including TKI therapy, CT, and radiotherapy—alongside IP treatment. Our study concluded that CT after LM (OR: 5.90; 95% CI: 1.46–23.88; *p* = 0.0129) and ECOG PS >2 (OR: 19.69; 95% CI: 1.76–220.03; *p* = 0.0155) are significant risk factors for severe myelosuppression. Although TKI after LM (OR: 5.15; 95% CI: 0.97–27.38; *p* = 0.0545) did not reach statistical significance, there was a trend toward increased risk. Consequently, undergoing additional systemic anti-tumor therapies—including TKI therapy and CT—alongside IP treatment may elevate the risk of myelosuppression, potentially explaining the higher incidence of myelosuppression observed in our study as compared to that in other studies.

In this study, BMI was not identified as a risk factor for myelosuppression following IP. This observation can be attributed to the characteristic behavior of most intrathecal chemotherapeutic agents, which do not rapidly transfer from the CSF to the bloodstream. The metabolic inactivation of these drugs in the CSF is negligible; instead, they are primarily eliminated directly from the CSF [28]. Considering the relatively constant volume of the subarachnoid space [29], the dose of intrathecal CT should be calibrated based on the CSF volume and drug concentration rather than on BMI.

The precise mechanism underlying myelosuppression induced by low-dose pemetrexed in IP treatment remains elusive. The pemetrexed dose utilized in IP typically ranges from 10 to 30 mg, which is substantially lower than the standard 500 mg/m² administered in intravenous CT. Nevertheless, myelosuppression persists as the primary adverse effect of IP. A plausible explanation involves the blood-brain barrier: the protein concentration in the CSF is significantly lower than that in the blood. This reduced protein-binding capacity of pemetrexed in the CSF may result in higher free-drug concentrations, potentially compromising bone marrow function.

Moreover, this study demonstrated that antiangiogenic therapy administered after LM significantly reduced the risk of severe myelosuppression following IP (HR: 0.23, 95% CI: 0.07–0.72; *p* = 0.0114). However, the precise mechanism through which antiangiogenic therapy mitigates the risk of severe myelosuppression post-IP remains elusive. Our previous research indicated that patients with LUAD-LM might benefit from a combination of osimertinib and bevacizumab. Specifically, bevacizumab significantly enhanced the intracranial concentration of osimertinib, suggesting that it may alleviate IP-induced myelosuppression by improving drug penetration through the blood-brain barrier and reducing pemetrexed accumulation in the CSF [30].

This study presents several limitations. First, as a retrospective analysis, the completeness and accuracy of the data relied on electronic medical records, which potentially introduced certain biases. Second, the study included patients treated at a single center, which may have resulted in selection bias. Third, the analysis did not include the dose and cycles of IP. This omission is due to the consistency of the IP regimen and dose in this study with current literature reports, where pemetrexed is typically administered at relatively low doses of 10–30 mg. Furthermore, the median cycles of IP prior to the development of myelosuppression and severe myelosuppression were 3 and 4, respectively, indicative of the induction phase of IP treatment. This study investigates myelosuppression development due to IP administration at a low dose, potentially offering insights into the prevention and management of IP-induced myelosuppression.

## 5 Conclusion

The present study identified age as a crucial risk factor for myelosuppression following IP, with a significantly elevated risk of severe myelosuppression in patients aged 58 years or older. These findings have substantial implications for IP administration guidelines, suggesting that IP should be administered cautiously in patients over 58 years of age, accompanied by the consideration of preventive strategies.

Subsequent research and more extensive prospective trials should prioritize the optimization of IP administration, frequency, and its integration with systemic therapies. Furthermore, investigating the underlying mechanisms of myelosuppression induced by low-dose IP treatment will be crucial for advancing this therapeutic approach.

## Data Availability

The dataset supporting the study’s conclusions is available from the corresponding author upon request, as its dissemination is limited by privacy and ethical considerations.

## List of Abbreviations

NSCLC: Non-small cell lung cancer
LM: Leptomeningeal metastasis
LUAD: Lung adenocarcinoma
IP: Intrathecal pemetrexed
CSF: Cerebrospinal fluid
OS: Overall survival
TKI: Tyrosine kinase inhibitors
BMI: Body mass index
ECOG PS: Eastern Cooperative Oncology Group Performance Status
BM: Bone metastasis
BMs: Brain metastasis
CT: Chemotherapy
WBRT: Whole-brain radiotherapy
ICI: Immune checkpoint inhibitors

## Appendix A

**Table A1:** Multivariate analysis for myelosuppression

Exposure	Non-adjusted		Adjust I		Adjust II	
	OR (95% CI)	*p*-value	OR (95% CI)	*p*-value	OR (95% CI)	*p*-value
**Age, year**	1.11 (1.03, 1.19)	0.0052*	1.11 (1.03, 1.19)	0.0053*	1.11 (1.03, 1.19)	0.0053*
**BMI, kg/m** ^**2**^	0.87 (0.73, 1.03)	0.1077	0.87 (0.73, 1.04)	0.1234	0.87 (0.73, 1.04)	0.1234
**BM**						
No	Refrence		Refrence		Refrence	
Yes	0.86 (0.26, 2.82)	0.8060	0.86 (0.26, 2.85)	0.8029	0.86 (0.26, 2.85)	0.8029
**ECOG PS**						
≤2	Refrence		Refrence		Refrence	
>2	inf. (0.00, Inf)	0.9903	inf. (0.00, Inf)	0.9904	inf. (0.00, Inf)	0.9904
**BMs**						
No	Refrence		Refrence		Refrence	
Yes	0.52 (0.13, 2.05)	0.3494	0.52 (0.13, 2.07)	0.3546	0.52 (0.13, 2.07)	0.3546
**CT after LM**						
No	Refrence		Refrence		Refrence	
Yes	3.05 (0.67, 13.83)	0.1491	3.05 (0.67, 13.83)	0.1487	3.05 (0.67, 13.83)	0.1487
**Antiangiogenic therapy after LM**						
No	Refrence		Refrence		Refrence	
Yes	0.18 (0.05, 0.61)	0.0062*	0.18 (0.05, 0.61)	0.0062*	0.18 (0.05, 0.61)	0.0062*
**TKI after LM**						
No	Refrence		Refrence		Refrence	
Yes	3.23 (0.67, 15.55)	0.1435	3.22 (0.66, 15.59)	0.1465	3.22 (0.66, 15.59)	0.1465
**WBRT after LM**						
No	Refrence		Refrence		Refrence	
Yes	0.91 (0.17, 4.86)	0.9078	0.91 (0.17, 4.86)	0.9084	0.91 (0.17, 4.86)	0.9084

Note: OR: odds ratio, CI: confidence interval, BMI: body mass index, BM: Bone metastases, ECOG PS: Eastern Cooperative Oncology Group Performance Status score, BMs: brain metastases, LM: leptomeningeal metastasis, CT: chemotherapy, TKI: tyrosine kinase inhibitor, WBRT: whole-brain radiotherapy, Model Ⅰ adjusted for age and sex. Model Ⅱ adjusted for age, sex, BMI, ECOG PS, BMs, BM, CT after LM, antiangiogenic therapy after LM, and TKI after LM. **p* < 0.05 indicates statistical significance.

## References

[1] Grossman SA, Krabak MJ. Leptomeningeal carcinomatosis. Cancer Treat Rev. 1999 Apr;25(2):103–19. 10395835 10.1053/ctrv.1999.0119

[2] Gleissner B, Chamberlain MC. Neoplastic meningitis. Lancet Neurol. 2006 May;5(5):443–52. 16632315 10.1016/S1474-4422(06)70443-4

[3] Wang Y, Yang X, Li NJ, Xue JX. Leptomeningeal metastases in non-small cell lung cancer: diagnosis and treatment. Lung Cancer. 2022 Dec;174:1–13. doi:10.1016/j.lungcan.2022.09.013; 36206679

[4] Ozcan G, Singh M, Vredenburgh JJ. Leptomeningeal metastasis from non-small cell lung cancer and current landscape of treatments. Clin Cancer Res. 2023 Jan 4;29(1):11–29. doi:10.1158/1078-0432.ccr-22-1585; 35972437

[5] Wu H, Zhang Q, Zhai W, Chen Y, Yang Y, Xie M, et al. Effectiveness of high-dose third-generation EGFR-tyrosine kinase inhibitors in treating EGFR-mutated non-small cell lung cancer patients with leptomeningeal metastasis. Lung Cancer. 2024 Feb;188(1):107475. doi:10.1016/j.lungcan.2024.107475; 38266613

[6] Felip E, Shaw AT, Bearz A, Camidge DR, Solomon BJ, Bauman JR, et al. Intracranial and extracranial efficacy of lorlatinib in patients with ALK-positive non-small-cell lung cancer previously treated with second-generation ALK TKIs. Ann Oncol. 2021 May;32(5):620–30. doi:10.1016/j.annonc.2021.02.012; 33639216

[7] Yang JCH, Kim SW, Kim DW, Lee JS, Cho BC, Ahn JS, et al. Osimertinib in patients with epidermal growth factor receptor mutation-positive non-small-cell lung cancer and leptomeningeal metastases: the BLOOM study. J Clin Oncol. 2020 Feb 20;38(6):538–47. doi:10.1200/jco.19.00457; 31809241 PMC7030895

[8] Zheng MM, Li YS, Jiang BY, Tu HY, Tang WF, Yang JJ, et al. Clinical utility of cerebrospinal fluid cell-free DNA as liquid biopsy for leptomeningeal metastases in ALK-rearranged NSCLC. J Thorac Oncol. 2019 May;14(5):924–32. doi:10.1016/j.jtho.2018.08.455. 30659989

[9] Li YS, Jiang BY, Yang JJ, Tu HY, Zhou Q, Guo WB, et al. Leptomeningeal metastases in patients with NSCLC with EGFR mutations. J Thorac Oncol. 2016;11(11):1962–9. doi:10.1016/j.jtho.2016.06.029; 27539328

[10] Wu YL, Zhou L, Lu Y. Intrathecal chemotherapy as a treatment for leptomeningeal metastasis of non-small cell lung cancer: a pooled analysis. Oncol Lett. 2016 Aug;12(2):1301–14. doi:10.3892/ol.2016.4783; 27446430 PMC4950629

[11] Pan Z, Yang G, Cui J, Li W, Li Y, Gao P, et al. A pilot phase 1 study of intrathecal pemetrexed for refractory leptomeningeal metastases from non-small-cell lung cancer. Front Oncol. 2019 Aug 30;9:838–49. doi:10.3389/fonc.2019.00838; 31544065 PMC6730526

[12] Pan Z, Yang G, He H, Cui J, Li W, Yuan T, et al. Intrathecal pemetrexed combined with involved-field radiotherapy as a first-line intra-CSF therapy for leptomeningeal metastases from solid tumors: a phase I/II study. Ther Adv Med Oncol. 2020 Jan;12:175883592093795–809. doi:10.1177/1758835920937953; 32733606 PMC7370561

[13] Li H, Zheng S, Lin Y, Yu T, Xie Y, Jiang C, et al. Safety, pharmacokinetic and clinical activity of intrathecal chemotherapy with pemetrexed via the ommaya reservoir for leptomeningeal metastases from lung adenocarcinoma: a prospective phase I study. Clin Lung Cancer. 2023 Mar;24(2):e94–104. doi:10.1016/j.cllc.2022.11.011; 36588048

[14] Fan C, Zhao Q, Li L, Shen W, Du Y, Teng C, et al. Efficacy and safety of intrathecal pemetrexed combined with dexamethasone for treating tyrosine kinase inhibitor-failed leptomeningeal metastases from EGFR-mutant NSCLC—a prospective, open-label, single-arm phase 1/2 clinical trial (unique identifier: ChiCTR1800016615). J Thorac Oncol. 2021 Aug;16(8):1359–68. doi:10.1016/j.jtho.2021.04.018; 33989780

[15] Fan C, Jiang Z, Teng C, Song X, Li L, Shen W, et al. Efficacy and safety of intrathecal pemetrexed for TKI-failed leptomeningeal metastases from EGFR+ NSCLC: an expanded, single-arm, phase II clinical trial. ESMO Open. 2024 Apr;9(4):102384–91. doi:10.1016/j.esmoop.2024.102384; 38377785 PMC11076967

[16] Zhou T, Zhu S, Xiong Q, Gan J, Wei J, Cai J, et al. Intrathecal chemotherapy combined with systemic therapy in patients with refractory leptomeningeal metastasis of non-small cell lung cancer: a retrospective study. BMC Cancer. 2023 Apr 11;23(1):333–44. doi:10.1186/s12885-023-10806-5; 37041504 PMC10088274

[17] Park K, Kim Y, Son M, Chae D, Park K. A pharmacometric model to predict chemotherapy-induced myelosuppression and associated risk factors in non-small cell lung cancer. Pharmaceutics. 2022 Apr 22;14(5):914. doi:10.3390/pharmaceutics14050914; 35631500 PMC9145791

[18] Han CJ, Ning X, Burd CE, Spakowicz DJ, Tounkara F, Kalady MF, et al. Chemotoxicity and associated risk factors in colorectal cancer: a systematic review and meta-analysis. Cancers. 2024 Jul 20;16(14):2597. doi:10.3390/cancers16142597; 39061235 PMC11274507

[19] Lyman GH, Abella E, Pettengell R. Risk factors for febrile neutropenia among patients with cancer receiving chemotherapy: a systematic review. Crit Rev Oncol Hematol. 2014 Jun;90(3):190–9. doi:10.1016/j.critrevonc.2013.12.006; 24434034

[20] Laskey RA, Poniewierski MS, Lopez MA, Hanna RK, Secord AA, Gehrig PA, et al. Predictors of severe and febrile neutropenia during primary chemotherapy for ovarian cancer. Gynecol Oncol. 2012 Jun;125(3):625–30. doi:10.1016/j.ygyno.2012.03.015; 22426251

[21] Le Rhun E, Weller M, Van Den Bent M, Brandsma D, Furtner J, Rudà R, et al. Leptomeningeal metastasis from solid tumours: EANO-ESMO clinical practice guideline for diagnosis, treatment and follow-up. ESMO Open. 2023 Oct;8(5):101624. doi:10.1016/j.esmoop.2023.101624; 37863528 PMC10619142

[22] Crawford J, Dale DC, Lyman GH. Chemotherapy-induced neutropenia: risks, consequences, and new directions for its management. Cancer. 2004 Jan 15;100(2):228–37. doi:10.1002/cncr.20218. 14716755

[23] Kuderer NM, Dale DC, Crawford J, Cosler LE, Lyman GH. Mortality, morbidity, and cost associated with febrile neutropenia in adult cancer patients. Cancer. 2006 May 15;106(10):2258–66. doi:10.1002/cncr.21847; 16575919

[24] Lyman GH, Michels SL, Reynolds MW, Barron R, Tomic KS, Yu J. Risk of mortality in patients with cancer who experience febrile neutropenia. Cancer. 2010 Dec;116(23):5555–63. doi:10.1002/cncr.25332; 20715160

[25] Zeiner PS, Filipski K, Filmann N, Forster MT, Voss M, Fokas E, et al. Sex-dependent analysis of temozolomide-induced myelosuppression and effects on survival in a large real-life cohort of patients with glioma. Neurology. 2022 May 17;98(20):e2073–83. doi:10.1212/wnl.0000000000200254; 35351796

[26] Hong Y, Miao Q, Zheng X , Xu Y , Huang Y, Chen S, et al. Effects of intrathecal pemetrexed on the survival of patients with leptomeningeal metastasis from lung adenocarcinoma: a propensity score matching analysis. J Neuro-Oncol. 2023 Nov;165(2):301–12. doi:10.1200/jco.2023.41.16_suppl.e21048. 37995007

[27] Geng D, Guo Q, Huang S, Zhang H, Guo S, Li X. A retrospective study of intrathecal pemetrexed combined with systemic therapy for leptomeningeal metastasis of lung cancer. Technol Cancer Res Treat. 2022 Jan;21:153303382210784–93. doi:10.1177/15330338221078429; 35289201 PMC8928347

[28] Fleischhack G, Jaehde U, Bode U. Pharmacokinetics following intraventricular administration of chemotherapy in patients with neoplastic meningitis. Clin Pharmacokinet. 2005;44(1):1–31. doi:10.2165/00003088-200544010-00001; 15634030

[29] Courchesne E, Chisum HJ, Townsend J, Cowles A, Covington J, Egaas B, et al. Normal brain development and aging: quantitative analysis at *in vivo* MR imaging in healthy volunteers. Radiology. 2000 Sep;216(3):672–82. doi:10.1148/radiology.216.3.r00au37672; 10966694

[30] Yi Y, Cai J, Xu P, Xiong L, Lu Z, Zeng Z, et al. Potential benefit of osimertinib plus bevacizumab in leptomeningeal metastasis with EGFR mutant non-small-cell lung cancer. J Transl Med. 2022 Dec;20(1):122. doi:10.1186/s12967-022-03453-0; 35287683 PMC8919569

